# Half Way to Hypusine—Structural Basis for Substrate Recognition by Human Deoxyhypusine Synthase

**DOI:** 10.3390/biom10040522

**Published:** 2020-03-30

**Authors:** Elżbieta Wątor, Piotr Wilk, Przemysław Grudnik

**Affiliations:** Malopolska Centre of Biotechnology, Jagiellonian University, ul. Gronostajowa 7a, 30-387 Krakow, Poland; elzbieta.wator@doctoral.uj.edu.pl (E.W.); wilk.piotr@uj.edu.pl (P.W.)

**Keywords:** hypusination, deoxyhypusine synthase, EIF5A, translation, hypusine, post-translational modification, polyamines, spermidine, spermine, putrescine

## Abstract

Deoxyhypusine synthase (DHS) is a transferase enabling the formation of deoxyhypusine, which is the first, rate-limiting step of a unique post-translational modification: hypusination. DHS catalyses the transfer of a 4-aminobutyl moiety of polyamine spermidine to a specific lysine of eukaryotic translation factor 5A (eIF5A) precursor in a nicotinamide adenine dinucleotide (NAD)-dependent manner. This modification occurs exclusively on one protein, eIF5A, and it is essential for cell proliferation. Malfunctions of the hypusination pathway, including those caused by mutations within the DHS encoding gene, are associated with conditions such as cancer or neurodegeneration. Here, we present a series of high-resolution crystal structures of human DHS. Structures were determined as the apoprotein, as well as ligand-bound states at high-resolutions ranging from 1.41 to 1.69 Å. By solving DHS in complex with its natural substrate spermidine (SPD), we identified the mode of substrate recognition. We also observed that other polyamines, namely spermine (SPM) and putrescine, bind DHS in a similar manner as SPD. Moreover, we performed activity assays showing that SPM could to some extent serve as an alternative DHS substrate. In contrast to previous studies, we demonstrate that no conformational changes occur in the DHS structure upon spermidine-binding. By combining mutagenesis and a light-scattering approach, we show that a conserved “ball-and-chain” motif is indispensable to assembling a functional DHS tetramer. Our study substantially advances our knowledge of the substrate recognition mechanism by DHS and may aid the design of pharmacological compounds for potential applications in cancer therapy.

## 1. Introduction

Hypusination is the post-translational modification of lysine to the unusual amino acid hypusine (N-ε-(4-amino-2-hydroxybutyl)lysine) [1]. This modification involves two enzymes, namely deoxyhypusine synthase (DHS) and deoxyhypusine hydroxylase (DOHH). In the first step of hypusination, the 4-aminobutyl moiety of spermidine (SPD) is transferred to the ε-amine group of lysine by DHS and forms deoxyhypusine, which is then hydroxylated by DOHH to hypusine. In humans, hypusine is present only in the eukaryotic translation factor 5A (eIF5A) [2,3]. Hypusination is the most specific post-translational modification known to date [4] and it is essential for eIF5A activity [5]. eIF5A is involved in elongation [6], termination [7] and the stimulation of peptide bond formation [8], and it facilitates protein synthesis by resolving polyproline-induced ribosomal stalling; thus, its role seems indispensable in proline repeat-rich protein synthesis [9]. The hypusination of eIF5A is essential for its activity in promoting cell proliferation [3,10,11,12]. Furthermore, there is evidence of eIF5A being important in different diseases such as diabetes, cancer and HIV infection [13,14,15,16,17]. In humans, there are two isoforms of eIF5A (eIF5A-1 and eIF5A-2) and both harbour hypusine modifications [18]. Despite the high conservation of the two isoforms, their functions may be significantly different [19]. The first isoform (eIF5A-1) is constitutively expressed in mammalian cells and overexpressed in human cancer tissues. In contrast to eIF5A, the expression of the other isoform (eIF5A-2) was detected only in specific tissues (testis and brain) or in various cancer types including colon, ovarian, bladder and liver [20]. Hypusinated eIF5A is essential for tumour growth, and its level of expression is closely related to the aggressiveness of cancers [21]. Moreover, the expression of the latter eIF5A-2 variant is a hallmark of many cancers [22,23].

DHS (EC: 2.5.1.46) is a cytosolic transferase involved in the first enzymatic step of the unique post-translational modification of eIF5A. In humans, DHS is encoded by the *dhps* gene, which is located on the 19th chromosome [24]. DHS is a 369 amino acid-long protein with a molecular mass of 41 kDa. In physiological conditions, DHS forms a homotetramer [25]. For its catalytic activity, the enzyme requires nicotinamide adenine dinucleotide (NAD) as a co-factor. DHS catalyses the transfer of 4-aminobutyl moiety of spermidine (SPD) to a specific lysine of eIF5A precursor, which results in deoxyhypusine formation. This reaction is the first, rate-limiting step of hypusination, and it plays a critical role in every eukaryotic living cell.

In detail, a DHS-catalysed reaction can be divided into four steps. During the first step, spermidine is oxidized in an NAD-dependent manner, which results in NADH and dehydrospermidine formation [26]. Generated dehydrospermidine is thereafter cleaved to yield diaminopropane (DAP), and the remaining butyloamine moiety is linked to the catalytic Lys329 via an imine bond [27]. This DHS-SPD intermediate is crucial for the entire reaction [28]. Upon eIF5A precursor recognition, the imine moiety is transferred to the ε-amino group of eIF5A Lys50. The last stage of the reaction catalysed by DHS is the reduction of the eIF5A Lys50 intermediate to deoxyhypusine through NAD regeneration [25].

Absent or diminished activity of DHS has a significant impact on cell proliferation [29,30]. At the beginning of 2019, mutations in the DHS-encoding gene were linked to a neurodevelopmental disorder, and a new hereditary recessive disease named ‘DHPS deficiency’ was described [31]. It has been also shown that the targeting of the first step of hypusination can suppress tumorigenesis [21]. The hypusination pathway can be inhibited by using a DHS inhibitor: N1-guanyl-1,7-diaminoheptane (GC7) [32,33], and this has shown promising results in the treatment of neuroblastoma [34] in combination with difluoromethylornithine (DFMO), which is a known inhibitor of ornithine decarboxylase [35]. However, GC7 is a spermidine-mimetic compound and thus, it may possibly also serve as an inhibitor of other SPD-binding proteins (e.g., spermine synthase), which is usually an undesired effect [13]. Therefore, the discovery of novel and more specific inhibitors is the current challenge of drug development.

Although the crystal structure of human DHS has been previously solved [36,37], its structure has been determined in either NAD- or GC7-bound states. Hence, we aimed to get molecular insights into the substrate-binding mechanism by solving DHS in the apo and polyamine substrate-bound states. Here, we report six high-resolution structures of wild-type (wt) human deoxyhypusine synthase, in the apo form and in complexes with polyamines, namely: spermidine, spermine (SPM) and putrescine (PUT). Our work presents the first structural insights into the substrate recognition and binding mechanism. Also, the structure of apo DHS reported herein is the first structure solved in the absence of the co-factor NAD. In addition, we demonstrate that a conserved, N-terminal “ball-and-chain” motif is essential for the assembly of the DHS homotetramer. Our research provides a solid basis for the development of specific DHS inhibitors, compounds which are likely to show significant anti-cancer activity.

## 2. Materials and Methods

### 2.1. Protein Expression and Purification

Genes encoding full-length DHS (Uniprot: P49366, residues 1–369) and a truncated DHS Δ1-52 (residues 53–369) were optimized for an *Escherichia coli* expression system, synthesized (Genescript) and cloned with an N-terminal 6xHis-tag followed by a TEV cleavage site into a pET24d plasmid using NcoI/BamHI restriction sites. *E. coli* BL21(DE3) cells were transformed with the pET-24d vector containing the appropriate sequence. Terrific Broth medium supplemented with kanamycin (50 µg/L) was inoculated at 1:100 with an overnight LB culture started from a single colony. After reaching OD_600_ = 1–1.2 the temperature was decreased from 37 °C to 18 °C, and IPTG was added to a final concentration of 0.5 mM. Protein expression was carried out while shaking at 210 rpm overnight. Cells were collected by centrifugation (17,700× *g*, 12 min, 4 °C) and re-suspended in lysis buffer (50 mM Tris-HCl pH 7.8, 300 mM NaCl, 20 mM imidazole, 10% glycerol, 10 mM β-ME). Cells were disrupted in the presence of lysozyme (Sigma-Aldrich) by sonication (15 min, 5 s pulse/3 s pause cycles). Homogenous cell solution, with the addition of benzonase (Sigma-Aldrich), was subjected to centrifugation (53,000× *g*, 45 min, 4 °C). After centrifugation, the cleared lysate was applied onto an equilibrated affinity column (5 mL HisTrap HP; GE Healthcare Europe GmbH, Freiburg, Germany) and washed with wash buffer (50 mM Tris-HCl pH 7.8, 200 mM NaCl, 40 mM imidazole, 5% glycerol, 5 mM β-ME) to elute non-specifically bounded proteins. The protein of interest was eluted with the elution buffer (50 mM Tris-HCl pH 7.8, 200 mM NaCl, 400 mM imidazole, 5% glycerol, 5 mM β-ME). Eluted protein was dialyzed against the storage buffer (50 mM Tris- HCl pH 7.8, 200 mM NaCl, 5 mM β-ME) and subjected to overnight TEV protease cleavage during the second step of dialysis to remove the affinity tag. After HisTag cleavage, Tag-free protein was separated from the undigested protein and HisTagged-TEV protease during the reverse HisTrap column chromatography. Fractions containing HisTag-free protein were concentrated using Amicon Ultra (Millipore) concentrator (cut-off: 10,000 kDa) and subjected to size-exclusion chromatography (SEC) on a HiLoad 16/60 Superdex 75 column in a storage buffer. Peaks of highest purity were pooled, concentrated, aliquoted and flash-frozen in liquid nitrogen for further analysis.

### 2.2. Protein Crystallisation

The initial screening for crystallisation conditions was performed using a variety of available commercial screens at Structural Biology Core Facility (www.structuralbiology.pl). The first crystals of DHS were obtained from a Morpheus grid screen [38] by the sitting drop vapor diffusion technique at 293 K and later further optimized by the fine tuning of each component concentrations. The DHS wt protein was concentrated to 20 mg/mL and mixed in a 1:1 ratio with mother liquor solution consisting of a 0.025–0.125 mM carboxylic acid mix, 30%–60% precipitant mix (MPD, PEG 1000, PEG 3350) and 100 mM Tris-Bicine with a pH of 8.5. Obtained crystals were transferred to the cryo-protectant solution containing 25% of ethylene glycol in mother liquor supplemented with the ligands of interest (50 mM SPD, SPM, PUT, NAD, DAP or lysine (LYS)), soaked for 1–5 min and flash cooled in LN_2_.

### 2.3. Diffraction Data Collection and Structure Determination

Diffraction data were collected at the MX-beamline 14.1 at the BESSY II electron storage ring (HZB, Berlin, Germany) [39]. The diffraction data were processed using XDS as implemented in the XDSAPP package [40]. The DHS crystal structures were solved by molecular replacement with Phaser [41] using 1DHS as a search model. The obtained models were rebuilt using Coot [42] and refined using Phenix [43]. During the refinement, ~1.5% of the reflections were used for cross-validation analysis to monitor the refinement strategy. Water molecules were automatically placed during structure refinement, or further added using Coot and subsequently manually inspected. The quality of the model was validated using MolProbity and PDB REDO [44] servers. All six structures have been determined to high resolution and refined to low values of R_work_ and R_free_, which are indicators of good accuracy of the models. For all structures, satisfactory geometrical parameters were obtained. All significant data collection, structure refinements and validation statistics are summarized in Table 1. The analysis and comparison of structures were performed in PyMOL (Molecular Graphics System, Version 2.0 Schrödinger, LLC).

### 2.4. Single Turnover Fluorescence Assay

The single turnover fluorescence assay was performed as described previously [45]. Briefly, 5 µM or 15 µM of DHS was incubated in the presence of 1 mM NAD (alone, or in the presence of 0.5 mM GC7 or 1 mM DAP) in 100 mM Tris-Bicine pH = 8.5 buffer, and the fluorescence excited at 350 nm and emitted at 441 nm was recorded. After ~ 2 min, SPD, SPM, PUT or LYS was added to a final concentration of 1 mM and measurement was immediately continued for ~ 2 min. An observed rapid burst of fluorescence, derived from a rising NADH concentration, was taken as the measure of DHS activity within the first step of its reaction. Each experiment was carried out at least three times.

### 2.5. Analysis of Protein Stability

To investigate the protein stability and determine the protein’s melting temperature, the Thermal Shift Assay (TSA) was performed as described previously [46]. Briefly, the protein solution (2 mg/mL) was incubated with 1:500 diluted Sypro Orange dye and storage buffer. The fluorescence signal (λ_ex_ = 492 nm, λ_em_ = 610 nm) from Sypro Orange was determined as a function of temperature between 5 and 95 °C in increments of 0.5 °C/min. The melting temperature was calculated as the inflexion point of the fluorescence curve. At least three independent repeats were done.

### 2.6. Analysis of the Protein Oligomeric State

The oligomeric states of DHS and DHS Δ1-52 were investigated by size-exclusion chromatography coupled to multi-angle light scattering (MALS), together with measuring the refractive index (RI) using the Dawn Heleos 8+ and T-Rex detectors (Wyatt). Pure protein sample was separated on the XBridge BEH200 7.8 × 300 (Waters) in 50 mM Tris H = 7.8, 200 mM NaCl, 5 mM β-ME buffer at a flow rate of 1 mL∙min^−1^. Results were analysed using the ASTRA6 software.

### 2.7. FRET Measurements

To investigate the DHS affinity to polyamine, a FRET experiment relying on the energy transfer from DHS W327 to the dihydronicotinamide ring of NADH was performed, as described previously [25]. Briefly, 5 µM of DHS wt was incubated in the presence of 10 µM NAD in 100 mM Tris-Bicine pH = 8.5 buffer, and sequentially, 2 uL of 50 µM SPD or 1 mM SPM were added to reaction mixture. After the addition of each substrate portion, the fluorescence signal (λex = 295 nm, λem = 441 nm) was recorded until saturation was achieved. Apparent K_D_ values were calculated using the Hill model implemented in Graphpad Prism 8 (GraphPad Software, Inc., California, USA).

## 3. Results

### 3.1. DHS Forms a Functional Tetramer

The structure of apo DHS was determined by molecular replacement using the Protein Data Bank (PDB) entry 1DHS as a search model and refined to a resolution of 1.52 Å with R_work_/R_free_ values of 15.42%/17.62%. In comparison, the previously reported structures have been refined at 2.2 and 3 Å for NAD- and GC7-bound structures respectively, thus the resolution of the structure presented by us constitutes a major improvement and allows for a more detailed analysis [36,37].

**Figure 1:**
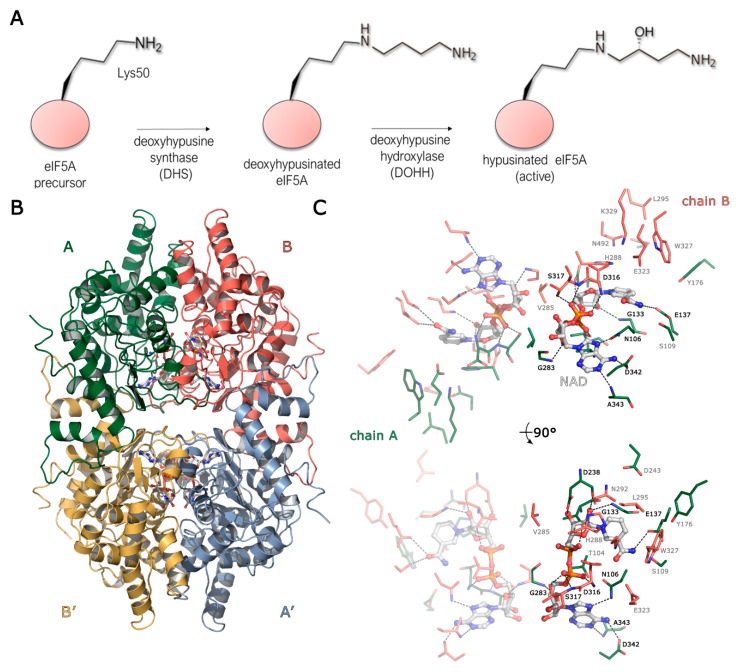
A deoxyhypusine synthase (DHS) overview. (**A**) A schematic representation of the hypusination pathway with DHS catalysing the first step, i.e., the transfer of the 4-aminobutyl moiety from spermidine (SPD) to a specific lysine side chain of eukaryotic initiation factor 5A (eIF5A) and deoxyhypusine hydroxylase (DOHH) finalising the modification by deoxyhypusine oxidation. (**B**) The structural organisation of the DHS tetramer. The tetramer is formed by two dimers with one (A-B, coloured green and red) observed in the asymmetric unit (ASU) of the crystal and the second dimer (A′-B′, coloured blue and yellow) observed by crystallographic symmetry. Ligands are shown as sticks, to highlight the locations of active sites. (**C**) A close-up view of the active sites formed at the dimer interface in the binary DHS-nicotinamide adenine dinucleotide (NAD) complex. Two NAD molecules are bound symmetrically and coordinated by residues from both subunits. The co-factor is shown as balls and sticks with carbon atoms coloured white, and residues from chains A and B are shown as sticks, with carbon atoms in green and red respectively. Residues binding the co-factor are black, and residues completing the active site are grey. For clarity, one of the active sites is semi-transparent.

DHS crystallised in the P3_2_21 space group with one tightly associated homodimer per asymmetric unit (ASU), with two active sites located at the dimer interface (Figure 1B). Each active site is formed by residues from both subunits. The DHS dimer is related by a two-fold axis with the identical dimer (Video S1), forming a dimer of dimers, which was previously identified as an active form of protein [47] and additionally confirmed by multiangle light scattering (MALS) measurements. In the refined structure, the electron density clearly defines the residues ranging from Ser28 to Met363. The loop containing residues 79–83 in chain A is poorly ordered compared to the rest of the protein, so these residues were not built due to insufficient definition by electron density. The core of DHS consists of five parallel beta-strands (S1, S2, S5, S6, S7) forming a Rossmann fold that is conserved in proteins that bind nucleotide co-factors (Figure S1B).

During the model building and structure refinement, we observed that Cys177, in both chains as well as in all of the herein presented structures, is covalently modified to a *β*-mercaptoethanol (BME) adduct. This may be irrelevant to structural studies, but it is noteworthy, particularly in light of the fact that in the absence of BME (or in the presence of another reductive agent as DTT), DHS does not crystallise, or it forms crystals characterised by poor morphology that are unsuitable for diffraction studies (Figure S1D).

Using PDBePISA [48] analysis, we calculated the total buried area of the apo DHS tetramer to equal 34,410 Å^2^, with a corresponding ΔG_int_ of −73.6 kcal/mol. The ΔG_int_ value indicates the solvation free energy gain upon the formation of the assembly. The possible dimeric assemblies formed by monomers A:B or A:B′ (where B′ is a symmetry-related chain) encompass a significantly smaller buried area and less favourable ΔG_int_ values of 9710 Å^2^/−18 kcal/mol and 10,320 Å^2^/−9.1 kcal/mol respectively. The same is true for all of the five complexes analysed in this work. This analysis suggests that the tetramer form is significantly more stable than any of the isolated dimers, and it is in agreement with the previous findings [49].

The DHS dimer present in the ASU has two major interfaces formed between chains A and B (A:B) and between chain A and a symmetry-related chain B (A:B′) (Figure 1B). The A:B interface of 2822 Å^2^ and ΔG_i_ of −21.4 kcal/mol involves 17 H-bonds and 16 salt bridges. This interface forms a binding pocket for NAD and SPD. The A:B′ interface of 2425 Å^2^ and ΔG_i_ of −33.9 kcal/mol is more hydrophobic in nature and encompasses only nine H-bonds and nine salt bridges. The diagonal chain interactions A:A′ and B:B′ play only a marginal role in complex formation with the interface’s area of approximately 400 Å^2^ and a ΔG_i_ of −4 kcal/mol.

The structure of human DHS in complex with NAD was previously reported by Liao et al. at 2.2Å, and the complex has been solved in the tetragonal space group with a single monomer in the ASU [37]. We have determined the structure of the DHS-NAD binary complex at 1.68 Å with R_work_/R_free_ values of 15.39%/16.62%. A comparison of both structures shows only small differences, with an overall CαRMSD of ~0.22 Å. Thus, we used the higher resolution structure for all further comparisons described herein. The electron density maps clearly show the presence of one NAD molecule bound in each of the active sites formed at the dimer interface (Figure 1C for details).

### 3.2. Crystal Structures of Binary DHS-Spermidine and Ternary DHS-Spermidine-NAD Complexes

**Figure 2:**
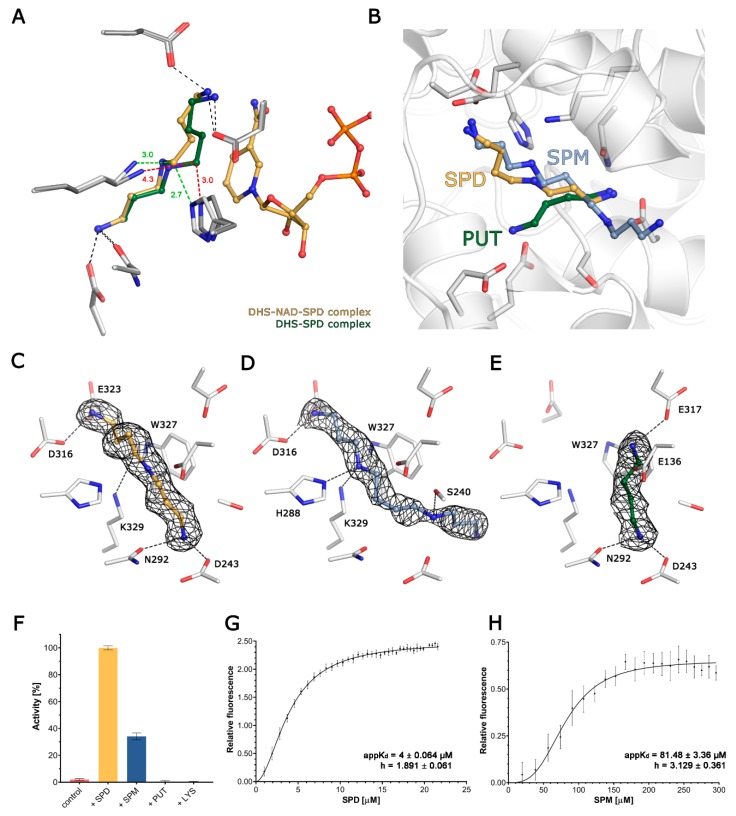
The binding of polyamines to DHS. (**A**) The superposition of binary DHS-SPD and ternary DHS-NAD-SPD complexes. The ligands are shown as balls and sticks, with carbons coloured green in binary complexes and yellow in ternary complexes respectively. The side chains for the residues coordinating SPD are shown as sticks, in light grey for DHS-NAD-SPD and in dark grey for DHS-SPD. The change in conformation of SPD induced by the presence of NAD is highlighted by a change of distances between C5 and His288 and the catalytical lysine 329, coloured red and green for binary and ternary complexes respectively. (**B**) Superposition of the DHS-SPD, DHS-SPM and DHS-PUT complex structures shows the relative positions of the investigated polyamines in the active site. Ligands are represented as balls and sticks, and the residues forming binding sites are shown as sticks. (**C**–**E**) The binding of SPD, SPM and PUT is shown with ligand as balls and sticks. 2Fo-Fc composite omit maps countered at 1σ are shown as a black mesh for each ligand. Residues taking part in the binding of each ligand are labelled. (**F**) The relative activity of DHS, expressed as an increase in the NAD fluorescence in the presence of various ligands. (**G**,**H**) A FRET binding assay for SPD and SPM.

To uncover the structural determinants of substrate recognition by DHS, we attempted to solve its crystal structure in the ligand-bound form. We have performed a number of crystal soaking experiments utilising a wide range of DHS substrates. First, we successfully soaked, measured the X-ray diffraction, solved and refined the structure of the DHS-SPD complex in the absence of the NAD co-factor. The structure was refined to a R_work_/R_free_ of 15.53%/16.22% at a 1.65 Å resolution. Subsequently, we solved and refined the structure of the DHS-NAD-SPD complex to a 1.41 Å resolution with R_work_/R_free_ values of 14.25%/15.98%. Both protein models encompass residues 8–363 of chain A and 28–363/364 of chain B, which all could be unambiguously fitted into the calculated electron density. In contrast to apo and NAD-bound structures, an additional N-terminal fragment containing regions from Glu8 or Ala9 is observed in chain A of spermidine bound complexes. The asymmetric unit of the structure accommodates two SPD molecules bound symmetrically to the two neighbouring active sites jointly created by two DHS monomers. It appears that in both binary and ternary complexes, the polyamine molecule is bound in the congenial orientation. The details of ligand binding are depicted in Figure 2A.

### 3.3. DHS Binds Spermine and Putrescine

To verify if the DHS active site is able to accommodate other physiologically relevant polyamines, we crystallised it in complexes with SPM and PUT. In the cellular context, tetraamine SPM is synthesised by spermine synthase from SPD and previous studies have shown no or weakly detectable binding of SPM by DHS [47]. We have determined a crystal structure of DHS-SPM complex at a 1.69 Å resolution. The final refined structure is characterised by R_work_/R_free_ values of 16.17%/17.93% respectively and accommodates residues 26–363 of chain A and 28–364 of chain B. Likewise, in the DHS-SPD complex, the electron density for two SPM molecules symmetrically bound could be observed (Figure 2D). In conclusion, the SPM binding mode is highly similar to that described for SPD.

Another physiologically relevant polyamine is PUT, a diamine synthesised in the polyamine synthesis pathway [50]. Ornithine and agmatine are precursors of PUT that are produced in ornithine decarboxylase (ODC)- or agmatinase (AGMAT)-catalysed reactions respectively. The PUT synthesis engaging arginase and ODC has been named the “classical route” in contrast to a so-called “non-classical route” that involves arginine decarboxylase and AGMAT [51]. In principle, PUT serves as the main precursor for spermidine synthesis in the reaction catalysed by spermidine synthase [52]. To test whether PUT is able to bind DHS, we crystallised and solved the DHS-PUT complex structure. The structure has been refined to 16.05%/17.46% at a 1.67 Å resolution. In both subunits present in the ASU, an additional electron density map was present in the active site, which was interpreted as PUT (Figure 2E).

Next, we also attempted to co-crystalize DHS in complex with 1,3-diaminopropane (DAP), which is the by-product of deoxyhypusine formation. However, our efforts resulted in an apo crystal structure with no observable electron density for a putative ligand.

### 3.4. Structural Comparison of Polyamine Binding by DHS

Based on our crystallographic studies, we show that three polyamines, namely SPD, SPM and PUT, but not DAP—which is a product of the DHS-catalysed reaction—can bind in the enzyme’s active site (Figure 2B). They all adopt an elongated conformation, yet display discrete differences which may discriminate between the substrate and its homologues. Notably, only minor differences in ADPs on the surface of the protein, and no significant structural changes, were observed between the compared binary structures outside the active site.

The physiological substrate, SPD, is anchored in the active site by its two terminal amine groups, with N1 forming a hydrogen bond with the side chain of D316, and N10 forming two H-bonds with the side chains of D243 and N292. The secondary amine (N6) is not involved in any hydrophilic interactions, but is stabilised by delocalised electrons from the nearby W327 (~2.7 Å). The distance from the ε-amine group of the catalytic K329 to SPD’s C5 is 4.23/4.22 Å in the binary complex, but is significantly shortened to 3.37/2.93 Å in the ternary one in the presence of NAD. Moreover, in the ternary (but not in the binary) complex, the C5 of SPD comes into close (2.75/2.65 Å) contact with the Nε of the H288, which may play a role in stabilising a transition state during the nucleophilic attack of SPD C5 on K239 Nε. Notably this orientation places the leaving group (DAP) close to the surface and facilitates its escape from the active site (Figure 2C).

SPM is similarly anchored by its N1 to the side chain of D316 and additionally to E323, but its chain is slightly bent in comparison to SPD due to the interaction of the secondary amine (N5, corresponding to C5 in SPD) with both K329 and H288, and these interactions prevent the occurrence of a nucleophilic attack (Figure 2D). The N10 in SPM (corresponding to N10 in SPD) maintains the interaction with D243, but instead of forming an H-bond with N292, it is shifted towards S240, forming a new H-bond. This interaction directs the remaining, uncoordinated part of SPM (C11-N14), to the active site exit. Noteworthily, despite the similar location of SPD and SPM in the active sites, both SPM molecules display significantly higher B-factor values as compared to SPD (Figure S2B–D).

PUT is a diamine that is significantly shorter than the other two investigated polyamines. In the active site, it is anchored by two H-bonds formed between N2 (corresponding to N10 in SPD) and the sidechains of D243 and N292. As it is shorter, the other end of PUT cannot reach the second anchoring point of SPD, but is rather deflected towards E136 and E137, forming salt bridges with their carboxylic groups. Hence, it is placed too distant to be involved catalytically with K329 (Figure 2E).

Surprisingly, based on the ΔG_i_ calculations for both SPD and SPM, in the range of 5–5.6 kcal/mol, and 1.9–2.2 kcal/mol for PUT, there is no clear energetic difference between the binding of either of the polyamines. The shortest tested polyamine, DAP, could not be observed in the crystal structures despite using very high concentrations during soaking or co-crystallisation, which is expected for the product of the reaction.

### 3.5. Spermidine and Spermine Cooperatively Bind DHS Active Sites

The first step of the reaction executed by DHS is the NAD-dependent oxidative cleavage of spermidine [26]. Thus, to further qualitatively assess the polyamine binding specificity, we used a well-established, single-turnover fluorescence assay. An observed rapid burst of fluorescence is a qualitative measure of NADH oxidation and reflects DHS activity. Surprisingly, not only the natural substrate SPD, but also one of related polyamines SPM, can serve as a reductive agent for NADH formation (Figure 2F). The fluorescence signal for SPM is significantly lower than that observed for SPD. At the same time, we observed no signal for the third human polyamine PUT or for LYS—the amino acid that undergoes modification catalysed by DHS. Using the same assay, we also tested if the product of further spermidine cleavage, DAP, is able to inhibit the reaction. Our results clearly show that the presence of DAP partially inhibits the reaction, but the inhibitory effect is significantly lower than that described for the only known DHS inhibitor: GC7 (Figure S2F).

**Figure 3:**
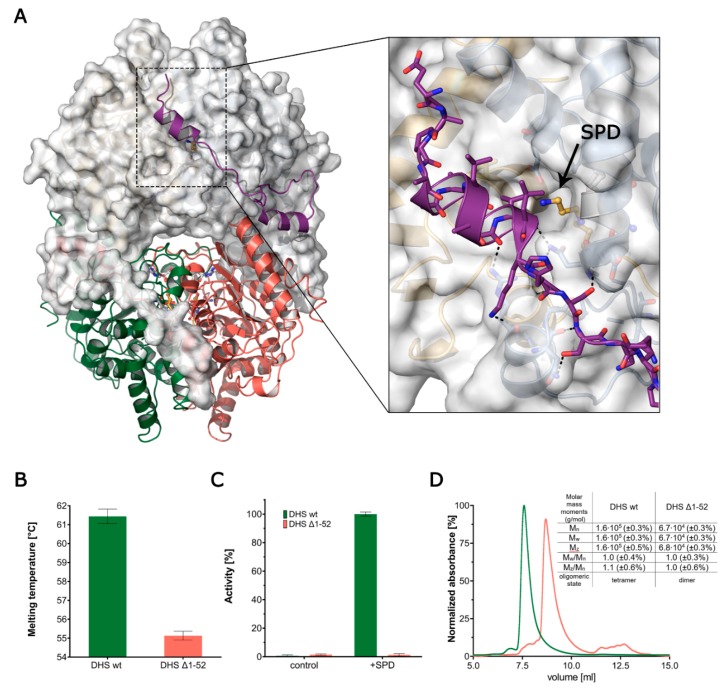
The role of the ball-and-chain motif. (**A**) The structure of DHS-NAD-SPD is shown with one dimer as a cartoon and one in surface representation. The ball-and-chain motif observed in subunit A is shown in purple. This region folds on the symmetry-related subunit over the entry to the active site. The enlargement shows the hydrogen bond network (black dashed lines) of the N-terminal fragment with the globular part of the enzyme. The opening of a tunnel, leading to the active site and a spermidine residue within, is indicated by an arrow. (**B**) The decrease of stability of the truncation mutant expressed as a drop in the measured Tm. (**C**) The relative activity of wild-type DHS and its truncation mutant. (**D**) The SEC-MALS elution profiles of wild-type and truncated DHS. The table summarises the numerical results of the MALS measurement. In all plots, green refers to the full-length DHS and red to the truncated DHS.

To compare the binding of SPD and SPM by DHS, we experimentally measured their apparent binding affinities using the FRET method, relying on the energy transfer from W327 to the dihydronicotinamide ring of NADH. We have analysed our data using the Hill slope binding model that accounts for DHS having four equipollent binding sites for polyamines. The calculated apparent K_D_ values for SPD and SPM were 4 ± 0.064 and 81.48 ± 3.36 µM, respectively (Figure 3F,G). The calculated Hill coefficient values (h) are 1.891 ± 0.061 and 3.129 ± 0.361, indicating positive cooperativity between binding sites.

### 3.6. The Ball-and-Chain Motif Is Crucial for DHS Activity, Stability and Oligomerisation

The high resolution of the presented structural data enables the tracing of an N-terminal ball-and-chain motif, which is comprised of a short helix connected to the core of DHS by a long flexible linker. The ball-and-chain motif encompasses residues 1–52 and contributes significantly to the protein stability by winding around a symmetry-related monomer (Figure 3A).

Based on the comparative analysis of the herein described structures, we noticed an increased flexibility of this motif, as indicated by higher-than-average ADPs and a lack of interpretable electron density in the subunit B (Figure S1B). The absence of the electron density for residues 8–27 in one of the chains can be either a mere crystallisation artefact or it can indicate that it is only partially ordered.

In the DHS-NAD-SPD complex, the A:B′ interface is 3092 Å^2^ and has a ΔG_i_ of −43.2 kcal/mol, of which as much as 1519 Å^2^ and −19.8 kcal/mol can be attributed to the aforementioned ball-and-chain motif [48]. In our structures, this ball-and-chain motif can be observed only in one chain in the asymmetric unit, and only for structures of complexes with SPD, and both SPD and NAD. To investigate the significance of this motif, we performed comparative studies between full length and truncated (Δ1–52) versions of DHS. The Thermal Shift Assay shows that a lack of this N-terminal part results in a significant decrease in protein stability from 61.5 ± 0.4 to 55.1 ± 0.4 °C (Figure 3B).

In the single turnover fluorescence assay performed for DHS Δ1-52, we observed an utter loss of spermidine dehydrogenation capacity, suggesting the importance of this motif for DHS functionality (Figure 3C). The absolute molecular weight measurements using MALS of both versions of the DHS protein show that without the ball-and-chain motif, the protein is not able to adopt a physiologically-active oligomeric state (Figure 3D). The elution time of DHS Δ1-52 indicates that the protein migrates in the form weighing approximately 68 kDa, which corresponds to the two monomers of the truncated version of the protein.

## 4. Discussion

Much attention is currently paid to the molecular mechanism of the selective control of protein translation by the hypusinated eIF5A. The DHS-catalysed reaction is a rate-limiting step in the hypusination pathway. Hence, a detailed knowledge of the reaction mechanism will help to understand the basics of this unique post-translational modification. Furthermore, since the hypusinated eIF5A is indispensable for tumour growth, as well as being closely related to the aggressiveness of cancers, the discovery of novel specific inhibitors of hypusination is highly awaited. Thus, the availability of high-resolution crystal structures in complex with natural ligands is essential to perform future structure-guided drug discovery attempts.

Low-resolution crystal structures of DHS have already been solved previously (PDB ID: 1RLZ, 1ROZ, 1RQD, 1DHS), yet our studies show not only more precise information due to significantly higher resolution, but also the enzyme in its apo form, as well as a detailed mode of binding of different polyamines. Previous findings suggested major conformational changes upon SPD and NAD binding; however, our structural data did not confirm this hypothesis (Figure S1B). Furthermore, it seems that substrate and co-factor binding influences only the active site of the protein and does not transmit any major conformational changes globally. Also, the study by Lee and Park proposes that SPD binding occurs following conformational changes induced by NAD binding [47]. Nonetheless, the DHS-SPD structure presented in our study challenges this suggestion, as SPD can bind DHS even in the absence of NAD. Structural studies on GC7-bound DHS underlined the importance of the His288 residue being a proton acceptor/donor during the DHS-catalysed reaction [36]. Our findings confirmed the proposed role of this invariant histidine. The comparison of binary DHS-SPD and ternary DHS-NAD-SPD complexes revealed an SPD conformational change, which is reflected in the change of distances between SPD C5, His288 and catalytical lysine 329.

To structurally describe substrate specificity, we determined the structures of DHS in complex with SPD and SPM. Both polyamines bind to DHS in the absence of NAD. Despite the almost identical mode of binding, only SPD serves as an appropriate DHS substrate. SPM activates DHS to a lesser extent than SPD, and its significantly higher flexibility probably excludes it as a substrate. Furthermore, our study refines a previously-proposed spermidine binding model. Briefly, we could observe the additional interaction of the SPD moiety with Asn292 and an additional stabilising hydrophobic interaction with NAD in the DHS-NAD-SPD complex.

Following the earlier reports on the role of PUT as a 4-aminobutyl acceptor, which essentially can substitute the eIF5A precursor, we successfully attempted to solve the DHS-PUT binary complex [53]. We observed that, to some extent, the PUT molecule accommodates an SPD binding site; however, as it is shorter than SPD, it is placed too distant to be involved catalytically with K329 (Figure 2E). This finding is also in agreement with the results of an activity assay that shows that, in contrast to SPD and SPM, PUT cannot serve as a reductive agent for NADH formation (Figure 2F).

As described in the introduction, the DHS-catalysed reaction can be divided into four steps, and previous studies demonstrated that the first two steps of the reaction, namely NAD-dependent spermidine oxidation and dehydrospermidine cleavage, take place even in the absence of the eIF5A [27]. It was speculated that possible reaction products could be 1,3-diaminopropane and Δ^1^-pyrroline, which follows the intermediate formation at the Lys329 residue. This reaction is believed to be insignificant in the physiological context [25]. Here we show that DAP inhibits the DHS-catalysed reaction; however, the inhibitory effect is significantly lower than that described for GC7 (Figure S2F). Furthermore, our studies exemplify the role of NAD in the stabilisation of a transition state during the nucleophilic attack of SPD C5 on K239 Nε. We could observe that the distance from the ε-amine group of the catalytic K329 to SPD’s C5 in the binary complex (DHS-SPD) is significantly shortened to in the ternary (DHS-SPD-NAD) one in the presence of NAD.

In the first study reporting the human DHS-NAD crystal structure, Liao and colleagues have attempted to use 1,7-aminoheptane, a SPD analogue, to uncover the structural determinants of substrate binding [37]. Nonetheless, they could not unambiguously locate this compound within the electron density in the active site. However, they reported a patch of continuous electron density found at the bottom of the inner pocket, which could not be explained by the 9-atom 1,7-aminoheptane moiety. The authors hypothesized that this density could be attributed to either a 4-aminobutyl moiety in a cyclised form (Δ-^1^-pyrroline 5-carboxylate) or as its straight-chain derivative. In the structure of DHS in complex with NAD, we also carefully inspected the calculated electron density. As a result, we have found an unexplained electron density localised at the DHS dimer-dimer interface. The above mentioned Δ-^1^-pyrroline 5-carboxylate fits well in this density, but does not entirely satisfy it. As we could not confirm its nature, therefore, we can only speculate that it may be a yet unidentified product of the DHS-catalysed reaction.

DHS forms an active homotetrameric assembly. Earlier studies identified a so-called ball-and-chain motif governing the entrance to an active site that hinders the substrate binding. Hence, it is believed that this N-terminal part of the protein undergoes a major conformational change upon both SPD and eIF5A binding. Furthermore, it has been shown that the deletion of this N-terminal part [54] inactivates DHS. Here, using the fluorescence activity assay and light scattering approach, we demonstrate that this N-terminal part of the protein (1–52) is indispensable not only for DHS activity but also for functional tetramer formation. Our results are in agreement with a previous report showing that the truncation of 48 N-terminal residues in yeast DHS inactivates the protein [54]. This inactivation may be not only associated with the role of the ball-and-chain motif in governing the active site entry, but also to the loss of the functional tetrameric conformation. The DHS active site is completely formed by two chains, so one may anticipate that the tetramer dissociation into dimers will not compromise the active site. On the other hand, dimer formation would also increase the solvent accessibility and hence it could lead to the destabilisation of the active site architecture. In consequence, with such an effect, the co-factor NAD may either adopt altered conformation or its binding could be compromised. Although this hypothesis is supported by the observed decrease in protein stability (Figure 3B), more extensive research, such as advanced quantum mechanics simulations studies, would be required.

The DHS structures reported herein lay the ground for the better understanding of substrate recognition, which may in turn be utilised in the design of novel pharmaceutics. However, cancer is not the only disease in which DHS plays a role. Recently, two mutations in the *dhps* gene were associated with the newly recognised DHS deficiency syndrome, manifested by a variety of clinical symptoms [31]. The first of the mutations leads to deletion of two amino acids, 305Y and 306I, located in one of β-strands forming the NAD binding Rossman fold. This deletion is likely to structurally destabilise and/or diminish the co-factor binding. The second mutation is an N173S substitution. Asparagine 173 is located at the entry of a tunnel connecting the SPD binding site with the protein exterior. Alterations in this region are likely to affect polyamine substrate/product exchange. More studies are needed to fully understand the impact of these mutations on the enzyme’s functionality.

## 5. Conclusions

In conclusion, our study, containing six novel high-resolution structures, provides a comprehensive framework for the future development of DHS-specific inhibitors. Notably, our findings close the remaining gap in the knowledge of substrate binding and recognition by DHS. Undeniably, future structural studies on eIF5A binding will shed more light on the DHS conformational changes during deoxyhypusine synthesis.

## Figures and Tables

**Table 1:** Data collection and refinement statistics.

DHS Structure	apo	NAD	NAD-SPD	SPD	SPM	PUT
PDB ID	6XXH	6XXI	6XXJ	6XXK	6XXL	6XXM
Wavelength (Å)	0.9184	0.9184	0.9184	0.9184	0.9184	0.9184
Resolution range (Å)	46.22–1.52 (1.57–1.52) *	46.09–1.68 (1.74–1.68)	46.24–1.41 (1.46–1.41)	46.19–1.65 (1.71–1.65)	46.08–1.69 (1.75–1.69)	46.12–1.67 (1.73–1.67)
Space group	P 3_2_ 2 1	P 3_2_ 2 1	P 3_2_ 2 1	P 3_2_ 2 1	P 3_2_ 2 1	P 3_2_ 2 1
Unit cell (Å,°)	104.97 104.97 161.03 90.0 90.0 120.0	104.82 104.82 160.50 90.0 90.0 120.0	105.49 105.49 160.83 90.0 90.0 120.0	104.98 104.98 160.90 90.0 90.0 120.0	104.90 104.90 160.43 90.0 90.0 120.0	104.73 104.73 160.67 90.0 90.0 120.0
Total reflections	1592643 (257143)	979651 (159039)	1946524 (308384)	1371934 (220304)	2283917 (365779)	1191506 (182975)
Unique reflections	157636 (15594)	116336 (18588)	198531 (19596)	123673 (12190)	114734 (11265)	118316 (11679)
Multiplicity	10.10	8.42	9.80	11.09	19.90	6.51
Completeness (%)	99.92 (99.88)	99.61 (98.77)	99.82 (99.48)	99.80 (99.05)	99.71 (98.40)	99.81 (99.45)
Mean I/sigma(I)	13.09 (0.76)	8.88 (0.92)	13.34 (0.76)	12.80 (0.94)	17.69 (0.75)	14.28 (0.95)
Wilson B-factor	22.41	26.10	20.06	26.06	31.73	25.73
R-merge (%)	10.6 (304.6)	12.6 (202.1)	8.8 (243.7)	11.0 (235.2)	10.5 (433.3)	10.6 (258.6)
R-meas (%)	11.1 (320.7)	13.5 (215.3)	9.4 (257.3)	11.6 (246.5)	10.8 (444.4)	11.2 (272.9)
R-sym (%)	10.6 (323.2)	12.5 (220.7)	9.1 (308.5)	11.3 (282.9)	10.8 (506.8)	10.8 (278.1)
CC_1/2_ (%)	99.9 (43.4)	99.6 (73.7)	99.9 (54.8)	99.9 (67.2)	100.0 (58.9)	99.9 (49.6)
Reflections used in refinement	157555 (15581)	116220 (11408)	198411 (19572)	123538 (12161)	114606 (11231)	118271 (11666)
Reflections used for R-free	2099 (208)	2097 (205)	2096 (207)	2098 (207)	2099 (206)	2098 (206)
R-work (%)	15.42 (34.35)	15.39 (34.91)	14.25 (34.82)	15.53 (36.48)	16.17 (39.78)	16.05 (33.51)
R-free (%)	17.62 (34.86)	16.62 (36.76)	15.98 (36.52)	16.22 (38.77)	17.93 (45.02)	17.46 (36.44)
Number of non-hydrogen atoms	6095	5916	6443	6013	6057	6186
macromolecules	5320	5317	5635	5439	5477	5596
ligands	22	243	215	169	107	20
solvent	753	356	593	405	473	570
Protein residues	667	679	692	691	675	692
RMS (bonds)	0.010	0.021	0.011	0.018	0.013	0.010
RMS (angles)	1.10	1.77	1.17	1.43	1.31	1.10
Ramachandran favored (%)	98.02	98.63	98.97	97.94	98.05	98.67
Ramachandran allowed (%)	1.98	1.37	1.03	2.06	1.95	1.33
Ramachandran outliers (%)	0.00	0.00	0.00	0.00	0.00	0.00
Rotamer outliers (%)	0.35	0.35	0.33	0.00	0.67	0.33
Clashscore	3.00	3.43	5.47	5.28	6.78	3.02
Average B-factor	34.78	39.05	29.52	36.93	47.86	38.77
macromolecules	32.88	37.87	27.97	35.76	46.59	37.79
ligands	51.62	54.97	44.86	55.55	69.85	55.71
solvent	47.71	45.85	38.63	44.94	57.59	47.81

* Statistics for the highest-resolution shell are shown in parentheses.

## Supplementary Materials

The following are available online at https://www.mdpi.com/2218-273X/10/4/522/s1, Video S1: Overall structure of DHS. Figure S1: Structural features of DHS. Figure S2: DHS substrate binding characteristics.
