# Supplementary material for: Half Way to Hypusine—Structural Basis for Substrate Recognition by Human Deoxyhypusine Synthase
Source: Biomolecules. 2020 Mar 30;10(4):522. doi: 10.3390/biom10040522 (PMC7226451; doi:10.3390/biom10040522)
Supplement: Supplementary file 1 [file biomolecules-10-00522-s001.zip › SUPPLEMENT_FIGURES.pdf]

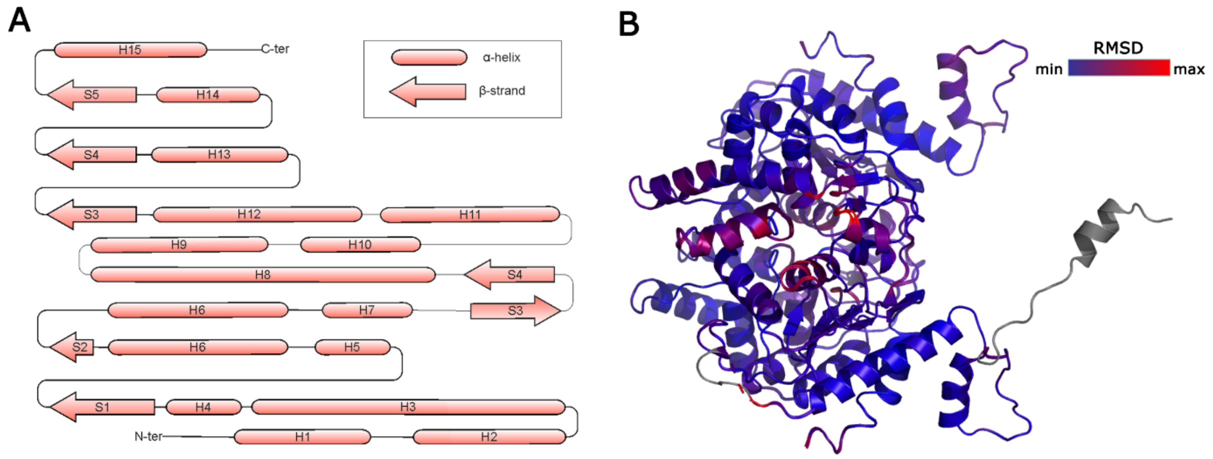

minimum / maximum / average distance between structures [Å]						
	apo	NAD	NAD & SPD	SPD	SPM	PUT
apo		0.02 /1.19/0.21	0.02/0.70/0.18	0.01/0.89/0.16	0.01/1.00/0.14	0.00/0.83/0.12
NAD	0.02 /1.19/0.21		0.01/1.29/0.12	0.01/1.58/0.15	0.01/0.98/0.17	0.01/1.33/0.16
NAD & SPD	0.02/0.70/0.18	0.01/1.29/0.12		0.01/0.92/0.13	0.01/1.59/0.17	0.02/0.79/0.15
SPD	0.01/0.89/0.16	0.01/1.58/0.15	0.01/0.92/0.13		0.01/1.81/0.10	0.01/1.01/0.09
SPM	0.01/1.00/0.14	0.01/0.98/0.17	0.01/1.59/0.17	0.01/1.81/0.10		0.00/0.80/0.09
PUT	0.00/0.83/0.12	0.01/1.33/0.16	0.02/0.79/0.15	0.01/1.01/0.09	0.00/0.80/0.09	

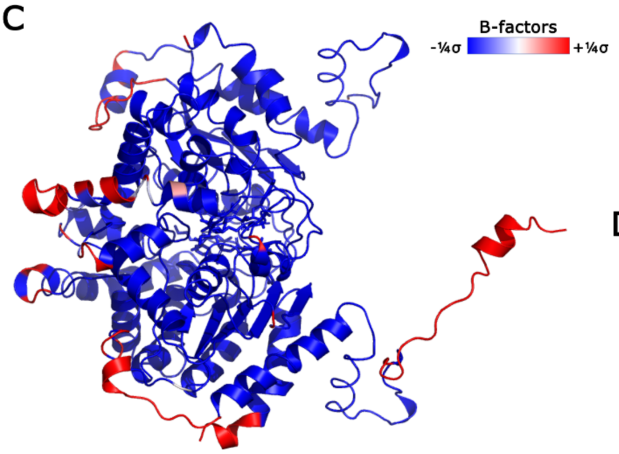

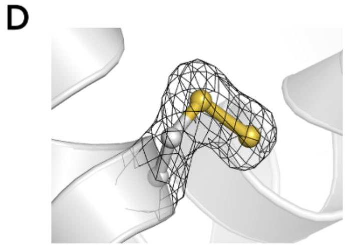

Figure S1. Structural features of DHS (A) Topology of DHS monomer. **(B)** Dimer of DHS-NAD-SPD (the content of ASU) coloured by its coordinates variability regarding DHS apo expressed as Calpha RMSD. Ball-and-chain motif is depicted in grey, and it is absent in the apo DHS structure. The table below summarizes the numerical values of Calpha RMSDs following pairwise comparisons of the different DHS complexes. **(C)** Dimer of DHS-NAD-SPD coloured by its B-factor values. **(D)** Representative 2Fo-Fc composite omit map countered at 1σ around one of the cysteine residues showing its modification by β-mercapthoethanol to S-mercaptocysteine (CSS).

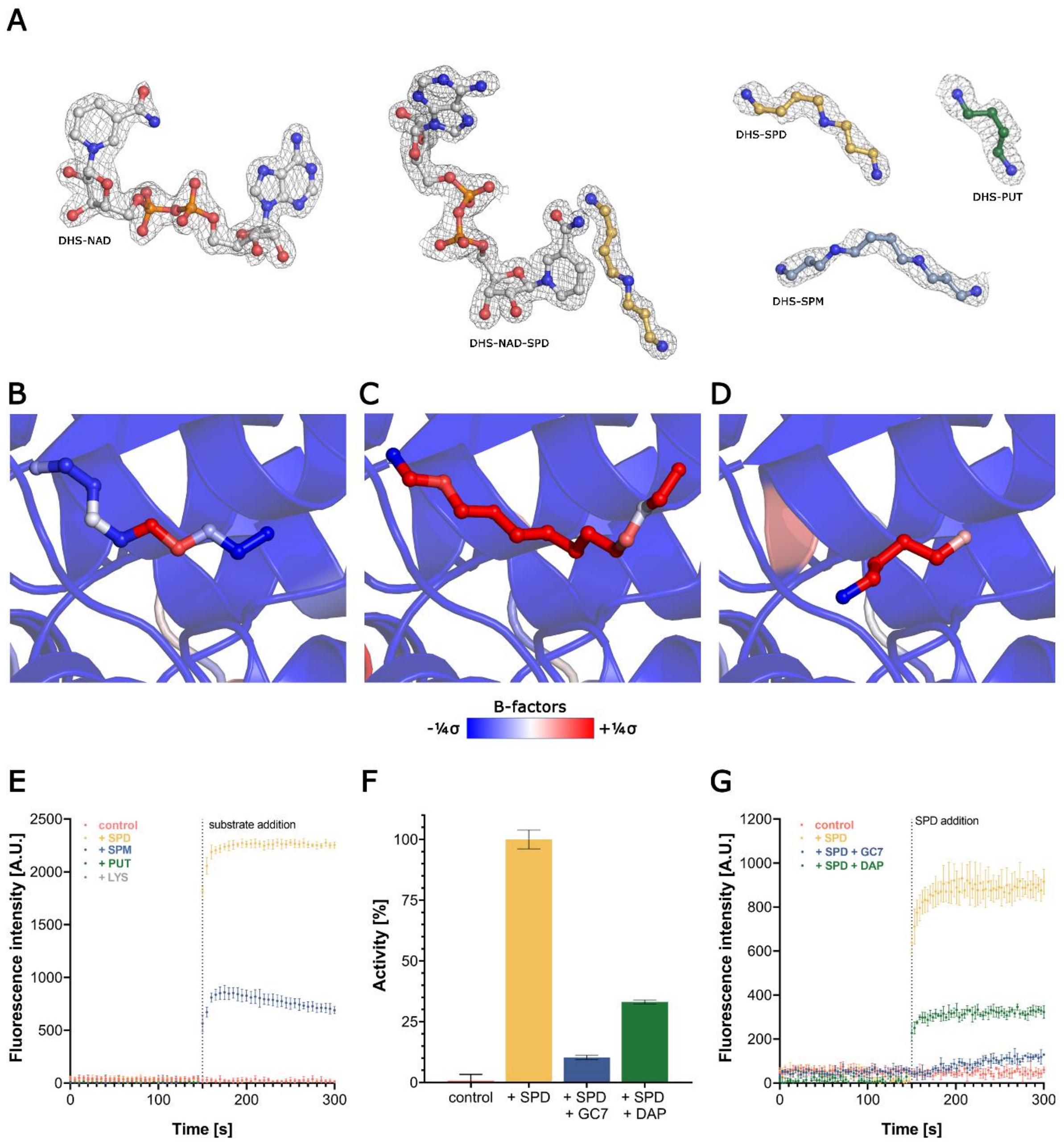

Figure S2. DHS substrate binding characteristics (A) 2Fo-Fc composite omit maps countered at 1σ level around the ligand molecules found in the described DHS-ligand complexes. Carbon atoms are coloured as follows: NAD in white, SPD in yellow, SPM in pale blue and PUT in green. **(B, C, D)** Differences in the ligand flexibility expressed by increased B-factor values. Panels are coloured in a blue-white-red scale based on the B average values +/- 1/4 standard deviation of B in a given structure. **(E-G)** Results of the single turnover fluorescence assay. E – the influence of selected potential ligands on NAD reduction by DHS. F, G – the influence of DAP (side-product) and GC7 (known inhibitor, substrate analogue) on the efficiency of DHS activation by SPD. The 4-fold decrease of fluorescence signal in the presence of DAP suggests a product inhibition.
